# Total knee arthroplasty in a patient with ipsilateral hip ankylosis: A case report

**DOI:** 10.1097/MD.0000000000016889

**Published:** 2019-09-06

**Authors:** XianZhe Tang, ZuYun Yan, Wanchun Wang, Tang Liu

**Affiliations:** Department of Orthopaedics, The Second Xiangya Hospital, Central South University, Changsha, Hunan, P.R. China.

**Keywords:** ipsilateral hip ankylosis, knee osteoarthritis, surgical position, total knee arthroplasty

## Abstract

**Rationale:**

There is a large number of people that have knee degeneration in China. Total knee arthroplasty is one of the most effective methods of treatment in the later stages of the disease. However, there are challenges when performing total knee arthroplasty on patients with ipsilateral hip akylosis. So far, there are few reports on postoperative curative effect of total knee arthroplasty for these patients. This case report records how to perform total knee arthroplasty in a patient with ipsilateral hip ankylosis.

**Patient concerns:**

Due to ankylosing spondylitis, the flexion of the patient's hips are restricted in 10°, which leads to a limited ipsilateral knee flexion to 30° when she is in the supine position.

**Diagnoses:**

Right knee osteoarthritis; right hip ankylosis.

**Interventions:**

We modified the traditional surgical position to allow easy exposure of the knee during surgery. After total knee arthroplasty, the patient was included in a planned training program, and was followed for 6 months.

**Outcomes:**

The patient walked well without ambulation aid and achieved satisfactory knee joint function.

**Lessons:**

Conversion of a fused hip to a total hip arthroplasty does improve the quality of life of patients, but, given the high incidence of complications and more financial burden to the patient, we modified traditional surgical position of the patient to provide ideal surgical exposure of the knee. We hope that this case can be used as a reference for clinicians to deal with similar situations.

## 1 Introduction

Total knee replacement is considered one of the most cost-effective treatments for final stage knee osteoarthritis to relieve pain and recover joint function.^[1–3]^ Many candidates for joint replacement have other joint lesions.^[4]^ The treatment challenges for total knee arthroplasty combined with hip ankylosis is how to solve the problem of limited knee movement during surgery. So far, there are few reports about the total knee arthroplasty in a patient with ipsilateral hip ankylosis and the postoperative curative effect. Our case report records how to conduct total knee arthroplasty in a patient with ipsilateral hip ankylosis who had ipsilateral hip fused in 10° of flexion and restricted knee flexion at 0° to 30°.

## 2 Case presentation

Our patient is a-64-year old Chinese female with knee pain of more than 10 years and limited activity for 7 years. The purpose of admission is to perform the right knee replacement to relieve pain and restore joint function. The patient suffered from chronic suppurative arthritis and chronic osteomyelitis caused by the right hip injury about 50 years ago, and she is currently suffering from right hip ankylosis. Eight years ago, she was treated with reduction and internal fixation of lumbar spondylolisthesis. The patient can walk independently without help, but is limited to 100 m due to knee pain. Many of her basic life activities cannot be completed, including wearing socks.

Physical examination: Right hip has sinus closure scar and hip joint fused in 15° of abduction, 15° of flexion, and 20° of external rotation and ipsilateral knee joint fused in 20° of flexion. There was tenderness in the medial joint space of the right knee and a sense of friction during activity. The patella grinding test was positive and patella lateral movement was less than 0.5 cm. Right knee range of motion was within 20° to 90° and friction fremitus was felt in the activity. Her right lower limb is 2 cm shorter than the left leg.

**Figure 1:**
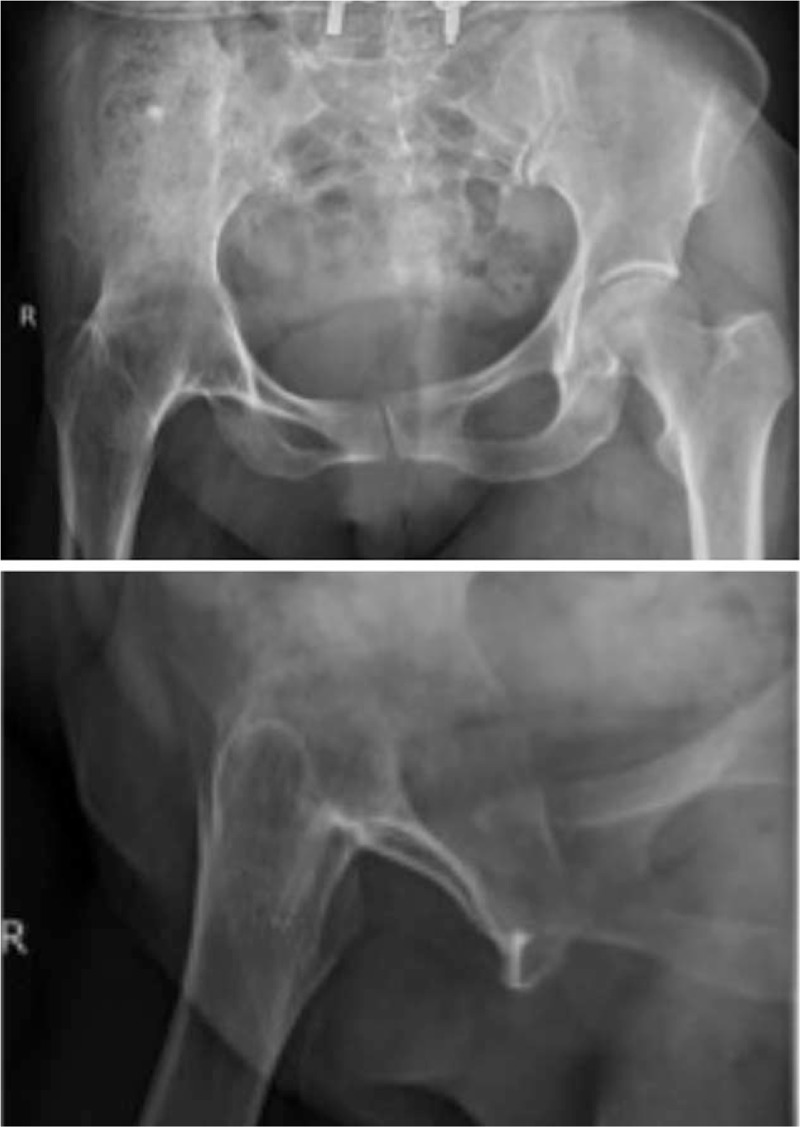
Recent X-ray of right hip joint. Shows the presence of her right hip joint fusion.

**Figure 2:**
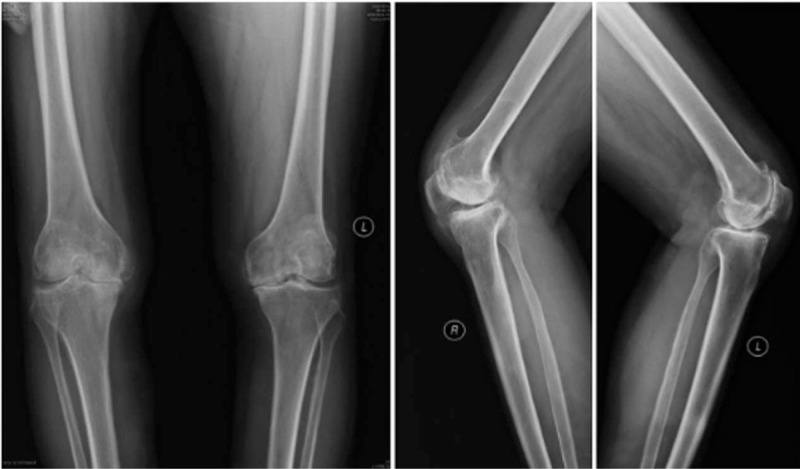
Preoperative radiographs of her knees. It suggests severe degeneration of knee joint and narrowing of knee joint space.

Preoperative radiographs: Her preoperative radiographs are as follows. Figure 1 shows the presence of right hip joint fusion. Figure 2 shows preoperative knee abnormality. Both her knees were severely degraded, and a large number of bone hyperplasia can be seen in the right medial knee.

**Figure 3:**
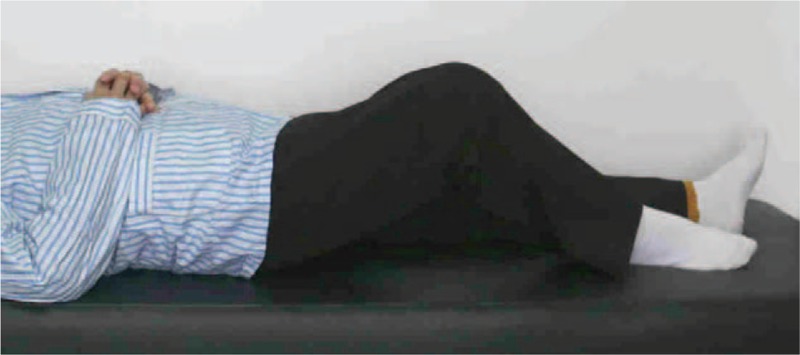
In the supine position, her hip joint fused in 15° of flexion, while the maximum flexion of the ipsilateral knee is only about 30°.

**Figure 4:**
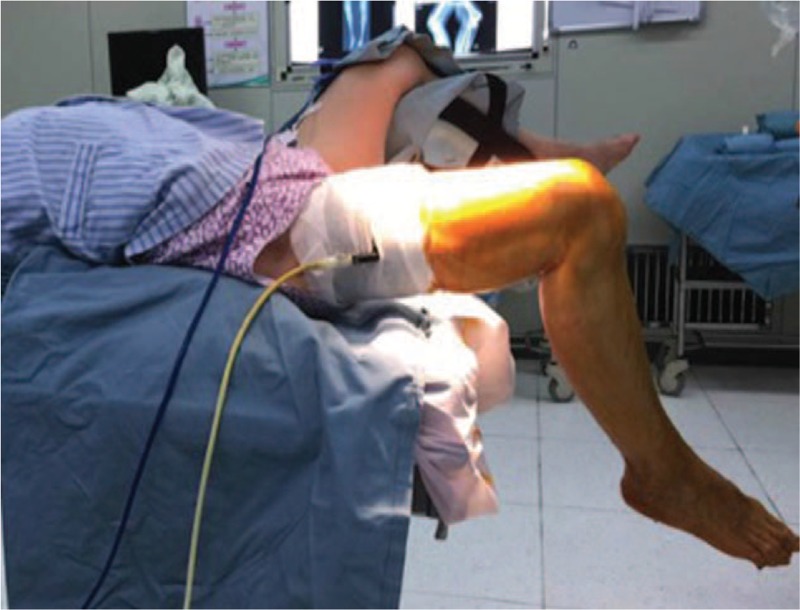
We modified position of the operation, this position allows the knee joint to achieve its maximum flexion in the operation.

**Figure 5:**
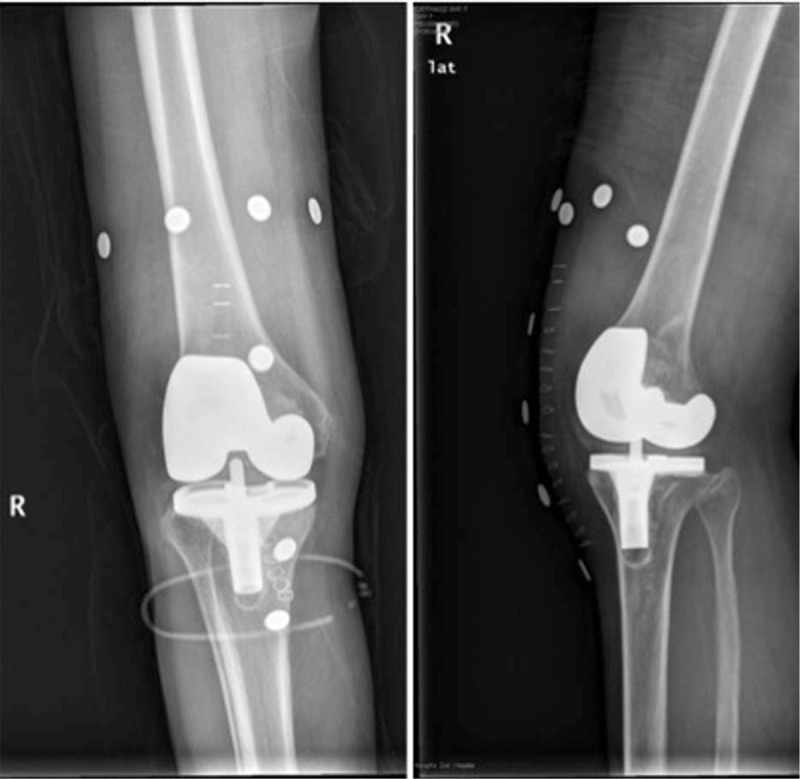
Postoperative check radiographs. Artificial knee prosthesis has been successfully implanted.

Methods (surgical technique): Knee arthroplasty with ipsilateral hip fusion faces difficulty in exposing the knee joint in the traditional supine position. In the supine position, the patient's hip joint is fixed at 15° of flexion, while the ipsilateral knee has a maximum flexion of only about 30° (Fig. 3). We took a special position: when the patient was placed on the operating table, the knee joint was just at the boundary between the tail plate and the back plate of the operating table, and the tail plate was removed to suspend the right leg at the end of the operating table. At the same time, in order to avoid interference with the operation, the lower limbs of the opposite side are placed at the lithotomy position. We took a surgical position similar to the lithotomy position to allow easy exposure of the knee during surgery (this position allows her right knee to flex 90° throughout the procedure) (Fig. 4). Figure 4 shows this position allows the knee joint to achieve its maximum flexion in the operation. Artificial knee joint prosthesis was implanted and postoperative X-ray showed that the joint prosthesis was firmly fixed (Fig. 5). As rehabilitation plays an important role in functional recovery after total knee arthroplasty,^[5]^ the patients was include in a rehabilitation plan focusing on range of motion of the knee after surgery.

**Figure 6:**
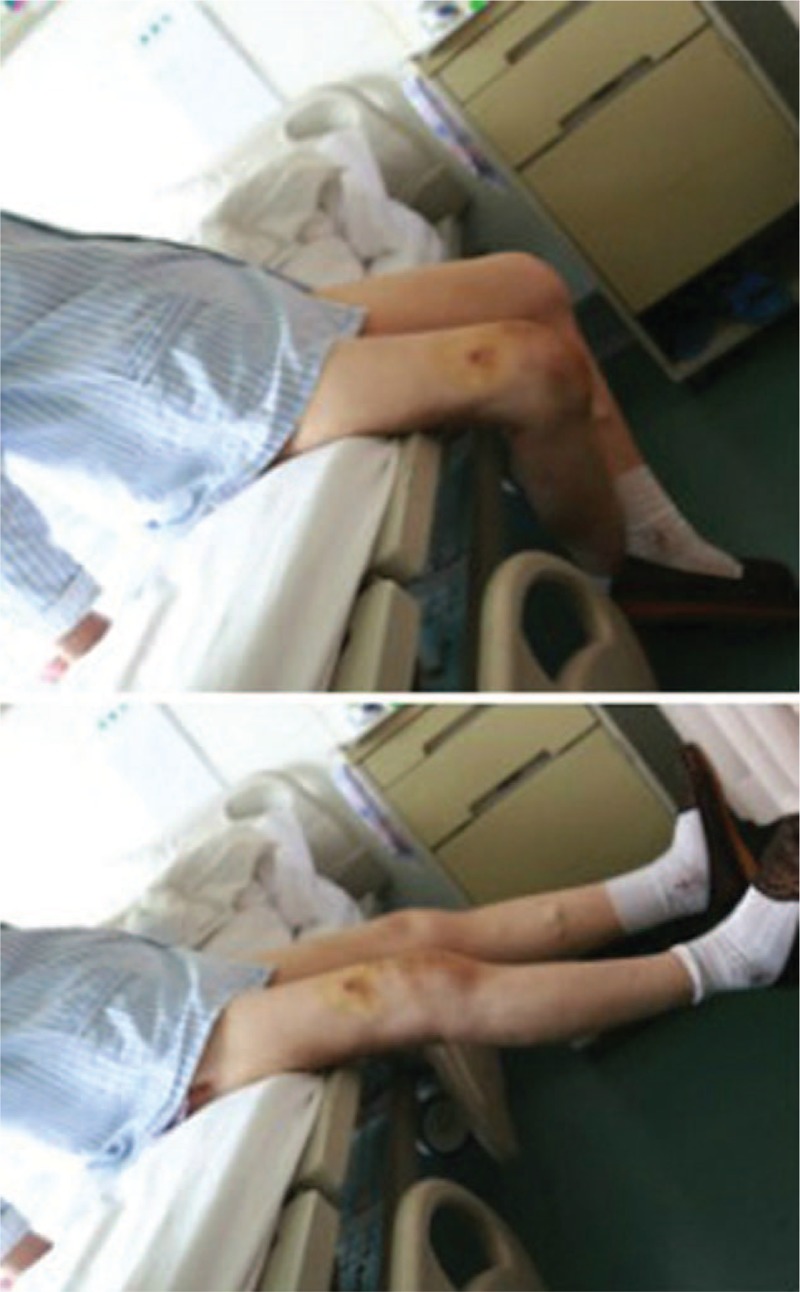
At 2 weeks postoperative, her right knee can basically be flexed to 90° and completely straight.

Outcomes and follow-up: The total follow-up period was 6 months. The patient was in good condition after surgery, and can walk with the help of ambulation aid on the 2nd postoperative day. Two weeks postoperatively, her right knee range of motion was at 0° to 90° (Fig. 6). At 3 months post-operative, her right knee range of motion was at 0° to 100°, and she walked well without ambulation aid.

## 3 Discussion

For total knee arthroplasty candidates who have ipsilateral hip arthrodesis, Romness et al and Rittmeister et al suggest the fused hip need to be made into a mobile joint before surgery.^[6,7]^ They conclude that performing total knee arthroplasty alone in patients with an ipsilateral fused hip ended in unsatisfactory results. However, their conclusion might be contributed into a lack of data. Conversion of a fused hip to a total hip arthroplasty does improve the quality of life of patients; however, a number of retrospective studies have shown that the conversion of hip arthrodesis to arthroplasty increases the risk of nerve injury and heterotopic ossification. Considering the possibility of a higher rate of complications in this specific situation, many data indicate that performing total hip arthroplasty is controversial, and may not be necessary if hip had been fused at an acceptable position.^[8–10]^

The technical challenge of knee arthroplasty for patients with ipsilateral hip fusion is how to solve the problem of limited knee movement during surgery. Koo et al wrote a case report and proposed a solution to overcome the challenge in the knee replacement in a patient with existing ipsilateral hip fusion.^[11]^ They changed the traditional supine position to allowed the knee to flex up to 100° on the operating table which originally can only flex up to 70° by elevating the fused hip joint. Goodman et al presented a different kind of body position to address the problem of exposure, especially in patients with the stiffness of the arthritic knee.^[12]^ By adjusting the height of the table tail episodically, they can change the flexion angle of the knee joint to achieve precise positioning and osteotomy. Comparing to Goodman et al's plan, Koo et al's plan had lager knee range of motion in the operation. While considering the effect of the stiffness of the arthritic knee, Goodman et al's approach has a more widely application. However, neither plan mentioned the placement of the contralateral lower limb.

How to solve the restriction of knee joint activity in operation is a technical problem when performing total knee arthroplasty in a patient with an ipsilateral fused hip. Conversion of a fused hip to a total hip arthroplasty does improve the quality of life of patients, but it accompany with high incidence of complications and more financial burden. We modified the patient position to facilitate operation process. However, the limitation of this study is that there is only 1 case and the follow-up time is not long enough. We hope that this case can be used as a reference for clinicians to deal with similar situations.

## Acknowledgments

The authors would like to thank this participating patient, as well as the study nurses, co-investigators, and colleagues who made this report possible.

## Author contributions

**Conceptualization:** Wanchun Wang, Tang Liu.

**Resources:** ZuYun Yan.

**Writing – original draft:** XianZhe Tang, ZuYun Yan.

**Writing – review and editing:** Tang Liu.
